# Case Report: Beyond type 1 diabetes: a case of delayed MODY1 diagnosis and successful transition to sulfonylurea therapy

**DOI:** 10.3389/fmed.2025.1590935

**Published:** 2025-05-23

**Authors:** Chiara Gulisano, Concetta Aloi, Alessandro Salina, Camilla Marazzi, Giordano Spacco, Serena Cappato, Renata Bocciardi, Dario Iafusco, Giacomo Tantari, Giuseppe d'Annunzio, Nicola Minuto, Mohamad Maghnie, Marta Bassi, Francesca Faravelli

**Affiliations:** ^1^Department of Neuroscience, Rehabilitation, Ophthalmology, Genetics, Maternal and Child Health (DINOGMI), University of Genoa, Genoa, Italy; ^2^Labsiem, Pediatric Clinic, Istituto di Ricovero e Cura a Carattere Scientifico (IRCCS) Istituto Giannina Gaslini, Genoa, Italy; ^3^Medical Genetics Unit, IRCCS Istituto Giannina Gaslini, Genoa, Italy; ^4^Regional Center for Pediatric Diabetes, Department of Pediatrics, University of the Study of Campania, Naples, Italy; ^5^Pediatric Clinic, IRCCS Istituto Giannina Gaslini, Genoa, Italy; ^6^Genomics and Clinical Genetics Unit, IRCCS Istituto Giannina Gaslini, Genoa, Italy

**Keywords:** MODY, HNF4A-MODY, MODY1, diabetes mellitus, continuous glucose monitoring (CGM), sulfonylureas

## Abstract

Maturity-onset diabetes of the young (MODY) is a rare, genetically heterogeneous form of diabetes characterized by early-onset dysglycaemia, typically before 25 years of age, and autosomal dominant inheritance. Among the different forms of MODY, HNF4A-MODY (MODY1) is caused by mutations in the *HNF4A* gene, which encodes a transcription factor essential for glucose metabolism. Here, we describe a novel splicing variant in the *HNF4A* gene (c.319+1G>A) identified in a 15-year-old girl with non-ketoacidotic diabetes and a family history of diabetes. Initially diagnosed with Type 1 diabetes (T1D), she required low insulin doses and displayed negative autoimmune markers. Genetic testing revealed the heterozygous variant inherited from her father and functional studies confirmed the variant's impact on splicing. Following the diagnosis of HNF4A-MODY, the patient's treatment was switched from insulin to sulfonylureas, resulting in improved glycaemic control and time in range, along with an improved quality of life. The report highlights the importance of considering MODY in young patients with diabetes who lack typical T1D characteristics and the value of combining clinical, genetic, and functional testing for accurate diagnosis and personalized treatment.

## 1 Introduction

Maturity-onset diabetes of the young (MODY) is an inherited form of non-autoimmune diabetes mellitus characterized by early onset dysglycaemia before 25 years of age, autosomal dominant inheritance, absence of β-cell autoantibodies (IAA, GADA, IA-2A, and ZNT8), and rare association with obesity (1, 2). MODY accounts for 1%−5% of all diabetes mellitus cases (3, 4) and is genetically heterogeneous, with 14 different causative genes identified to date. Among these, HNF4A-MODY (MODY1) (OMIM #125850) accounts for 10% of cases in the European MODY population (5). HNF4A-

MODY is caused by pathogenic defects in the hepatocyte nuclear factor 4 alpha gene (*HNF4A*) (OMIM #600281), which encodes for the HNF4A transcription factor. HNF4A is essential for glucose transport and metabolism, and is expressed predominantly in the liver, pancreatic islets cells and kidney (2, 6). Variants in the *HNF4A* gene cause a reduction in insulin secretion. Sulfonylureas are effective in managing patients with HNF4A-MODY (7). With the growing number of variants detected by NGS, the availability of reliable functional assays is essential to evaluate their impact on gene function and expression in order to provide patients with the proper diagnosis and management.

We describe a novel splicing variant of the *HNF4A* gene identified in a familial case of MODY and its functional characterization, enabling a diagnosis of MODY1 in a teenage patient.

## 2 Case description

A 15-year-old girl was diagnosed as diabetes mellitus (DM) with non-ketoacidotic onset: fasting hyperglycaemia 379 mg/dl, HbA1c 8.57% (70 mmol/mol), C-peptide 1.96 ng/ml, normal pH, absence of ketonemia, polyuria, or polydipsia. Antibodies against pancreatic beta cells (anti-IA2, anti-ZnT8, anti-GAD, and anti-insulin) were negative.

She was born at term of an uneventful pregnancy, with an appropriate weight for gestational age at birth. No perinatal issues, in particular no hypo- or hyper-glycaemia, were reported. Her medical history was unremarkable. Family history was positive for diabetes mellitus: the father with insulin-treated Type 2 Diabetes Mellitus (T2DM) and paternal grandfather with unspecified DM.

**Figure 1:**
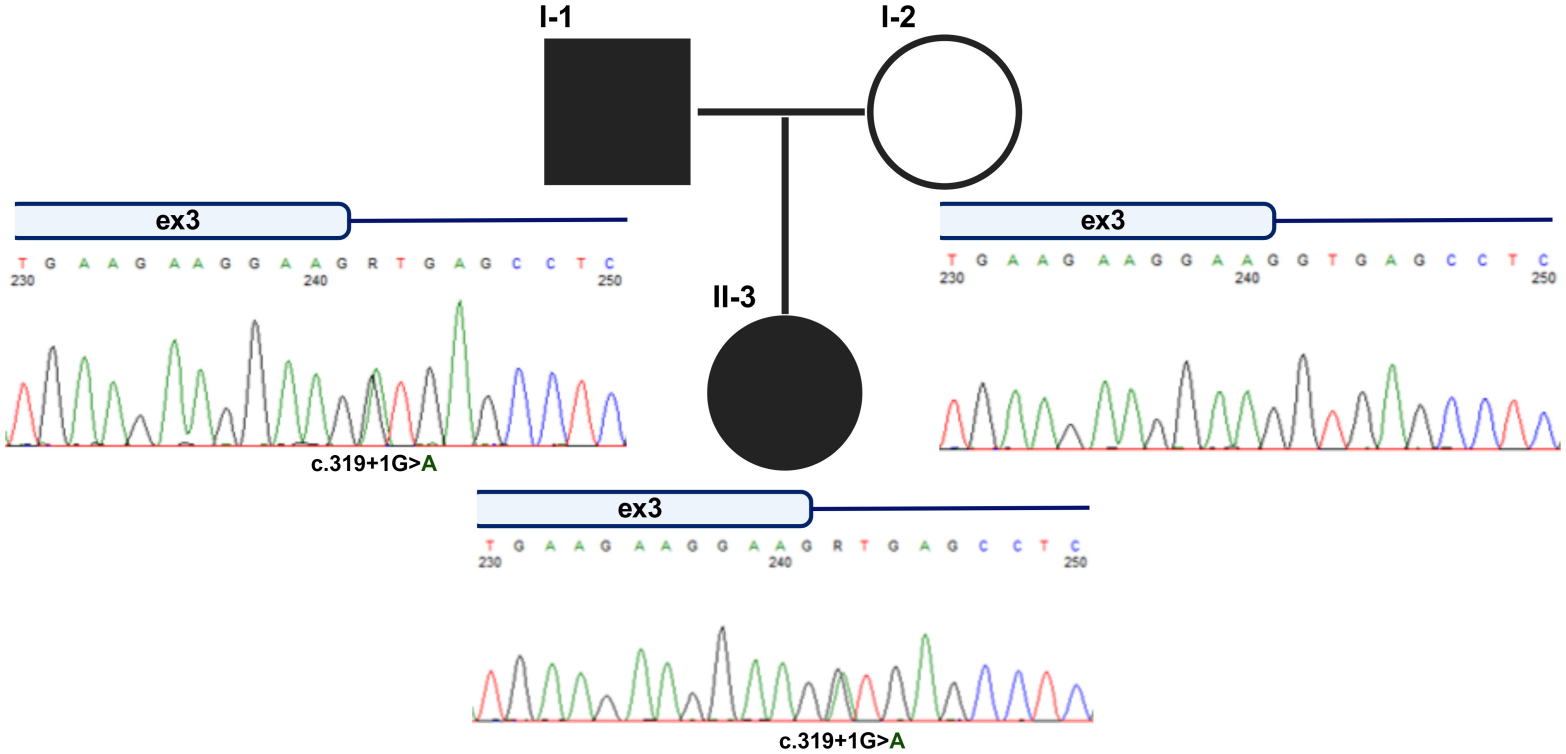
Pedigree of proband and segregation analysis of the novel *HNF4A* variant. Sanger sequencing was used to validate the presence of the novel *HNF4A* variant identified by NGS and to verify the segregation in the proband's family. As shown by the chromatograms, the substitution was inherited from the affected father.

Multi-injection insulin therapy, adapted on continuous glucose monitoring (CGM), was prescribed and the patient was assigned a provisional diagnosis of Type 1 Diabetes (idiopathic, absence of autoimmunity). Due to a persistent low insulin requirement (0.3 U/kg/day), autoimmune testing was repeated 1 year later and resulted negative. In the light of this result and the positive family history of DM, a genetic investigation for monogenic diabetes (i.e. Maturity Onset Diabetes of the Young, MODY) was performed (8). Next-Generation Sequencing (NGS) analysis involving 45 genes associated with dysglycaemia revealed a heterozygous variant (c.319+1G>A) in intron 3 of the *HNF4A* gene (Figure 1). The variant, inherited from the father, was predicted to impact splicing as confirmed by functional studies. Based on these results, a therapeutic switch to sulfonylureas (SU) was proposed to the patient, resulting in good control of her glycaemic levels.

## 3 Diagnostic assessment

### 3.1 Molecular diagnosis

Genomic DNA was obtained from PBMC of the proband and her father upon administration of an informed consent. Molecular screening was performed by analyzing a panel of 45 genes involved in monogenic diabetes and dysglicaemia (Supplementary Table 1) filtered from whole Exome Sequencing (ES) data (9). 50–100 ng of genomic DNA was used to sequencing selection of coding genomic regions and flanking intronic sequences using IDT xGen Exome Research Panel v2 enrichment kit (34 Mb, 19,433 genes) and Illumina technology (PE 2X150) on the Illumina platform Novaseq6000. Bioinformatics workflow for variant calls based on GATK software.

The patient was found to be heterozygous for a novel, intronic variant of the *HNF4A* gene (OMIM# 600281), the c.319+1G>A (p.°?) (RefSeq, NM_175914.5, and NP_787110.2), nomenclature according to the *HGVS* guidelines. The presence of the variant was confirmed by direct Sanger sequencing. Briefly, PCR products, corresponding to the genomic region of interest, were purified by enzymatic digestion with Exo/SAP-IT (Thermo Scientific^®^, Massachusetts, USA) and sequenced with the Big Dye Terminator Cycle Sequencing Kit (Thermo Scientific^®^, Massachusetts, USA) according to the provided protocol; sequencing reactions were run on a 3,130 × l Genetic Analyzer (Thermo Scientific^®^, Massachusetts, USA) and analyzed with the Sequencer 4.7 software (Genecodes^®^, USA).

### 3.2 Generation of the minigene constructs

The effect of the *HNF4A* variant on the mRNA splicing was verified by the minigene approach, based on pSPL3 exon trapping vector (already available in the Lab) (10).

A genomic fragment of 450 bp (chr20:44,407,193–44,407,642; GRCh38/hg38) spanning the third coding exon of *HNF4A* and flanking intronic sequences was amplified by PCR from genomic DNA of the proband. Wild-type and variant allele were separated by subcloning in pCR2.1-TOPO TA (TOPO™ TA Cloning, Invitrogen, Thermo Fisher Scientific, Massachusetts, USA), checked by Sanger sequencing and then subcloned into the pSPL3 splicing vector for functional analysis.

### 3.3 Cell culture, transient transfection and minigene sequences analysis

The Hek-293 cell line was already available in the laboratory and previously purchased by ATCC. Cells were routinely cultured in complete medium consisting of Essential Modified Medium (MEM) with 10% FBS in a humidified incubator at 37°C with 5% CO_2_. Transient transfections for the minigene assay were carried out by seeding 8 × 10^5^/well cells in 6-well plates. The next day, the transfection mix composed by 2 μg of pSPL3 constructs and Lipofectamine 2,000 reagent was added to cells, as suggested by the manufacturer (Invitrogen^®^, Thermo Fisher Scientific, Massachusetts, USA). Cells were then collected after 24 h and processed for RNA extraction with the RNeasy plus Mini Kit (Qiagen^®^, Hilden, Germany) according to the protocol provided. cDNA was obtained by the retro-transcription of 1 μg of RNA by using Advantage^®^ RT-for-PCR (Takara, Shiga, Japan) and then, amplified with GoTaq Master mix (Promega^®^, Wisconsin, USA) as indicated by the manufacturer's protocol (oligonucleotides sequences are available upon request). The PCR products were checked on 1.5% Agarose gel, cleaned-up by Exo/SAP-IT (Thermo Scientific^®^, Massachusetts, USA) digestion and sequenced as described above.

### 3.4 Results

The identified *HNF4A* variant, inherited by the proband from the affected father (Figure 1), has never been reported in association with MODY and is absent in population databases. According to the ACMG criteria the variant is interpreted as likely pathogenic (PVS1, PM2) (11). This substitution affects the canonical donor site of the *HNF4A* exon 3 and is predicted to alter the splicing with a significant Δ score of 0.95 (high precision) by the splice AI bioinformatic tool (12), thus prompting us to proceed with the experimental validation by a minigene approach (10).

**Figure 2:**
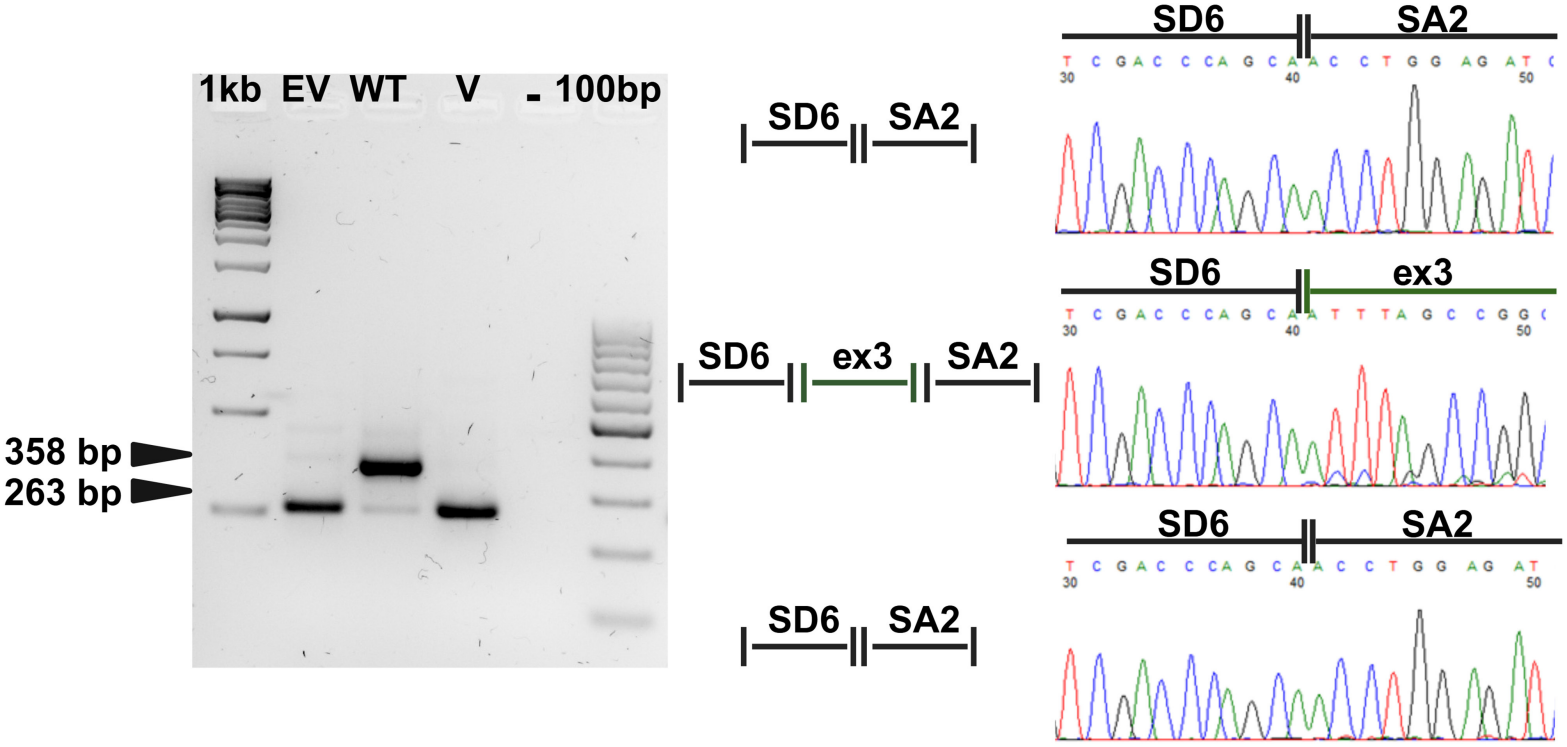
The novel *HNF4A* variant affects the splicing of the gene. RT-PCR products obtained from Hek-293 cells transiently transfected with the different pSPL3 constructs. A band of 263 bp was detectable both in cells transfected with the empty vector (EV) and with the construct carrying the third *HNF4A* exon 3 and flanking intronic sequences with the c.391G>A variant (V). This is due to the skipping of the *HNF4A* exon induced by the presence of the substitution with combination of the two artificial exons as observed in cells expressing the empty vector. A band of 358 bp is detectable in cells expressing the wild-type minigene construct (WT) and corresponding to the correct splicing combining the exon 3 of the *HNF4A* gene with those provided by the splicing vector, as schematically represented in the middle panel. The splicing events were also checked by Sanger sequencing of the RT-PCR products as shown by the chromatograms reported in the right panel. 1kb and 100bp, molecular weight markers; -, PCR reaction negative control; SA and SD6, artificial exons provided by the pSPL3 vector.

As shown in Figure 2, RT-PCR amplification products with different sizes were detected in cells expressing both wild-type (WT) and mutated pSPL3 constructs. Sanger sequencing allowed us to verify that the bands migrating with lower molecular weight corresponded to what expected by the correct splicing of the vector exons in cells transfected with empty vector, and to an altered splicing with skipping of the *HNF4A* exon 3 in cells transfected with the mutated construct. A proper splicing combining vector exons with *HNF4A* exon 3 was detectable in cells expressing the WT construct. These results demonstrated that the variant alters the *HNF4A* splicing by causing the exclusion of the exon 3 from mature mRNA. This exon is out-of-frame and its skipping from cDNA leads to a frameshift with generation of an early termination codon, likely triggering the nonsense-mediated mRNA decay (NMD) of the mutated transcript. Splicing alterations of *HNF4A* are a known pathogenic mechanism in MODY1 (13).

### 3.5 Clinical management

Based on the confirmed molecular diagnosis of *HNF4A*-MODY1, we planned a therapeutic switch to sulfonylureas (SU), which would likely improve the patient's quality of life and glycaemic control. The therapeutic switch was conducted as follows: on Day 1, therapy with Gliclazide started at a dosage of 30 mg per day, pre-prandial insulin boluses were suspended and the dosage of basal insulin was halved. On Day 2 basal insulin was discontinued and CGM showed initially optimal glycaemic values. No episodes of hypoglycaemia occurred.

The patient was initially followed-up monthly in the outpatient Clinic. At the first follow-up visit, isolated postprandial hyperglycaemic peaks, consistently below 250 mg/dL, were reported and Gliclazide dosage was increased to 60 mg per day. The glycaemic trend remained stable. Throughout the follow-up, screening for DM complications was performed according to ISPAD guidelines and no complication occurred (14).

**Figure 3:**
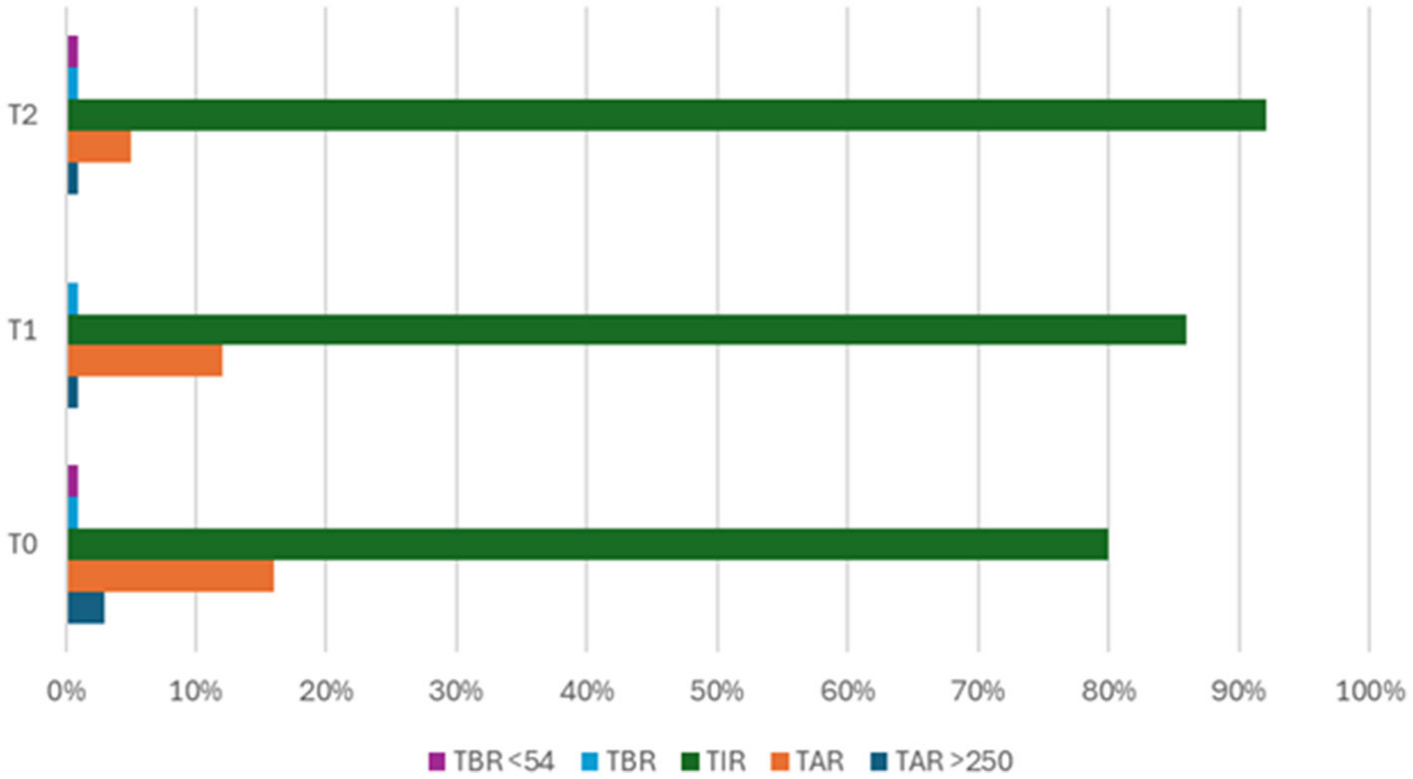
Glycaemic improvement after starting Gliclazide. TAR>250, Time Above Range >250; TAR, Time Above Range; TIR, Time In Range; TBR, Time Below Range; TBR <54, Time Below Range <54; T0, in the course of insulin therapy; T1, after starting Gliclazide; T2, Gliclazide dosage stabilized at 60 mg per day.

In Figure 3, we summarized CGM parameters at T0 (during insulin therapy), at T1 (after starting Gliclazide), and at T2 (at the last follow-up visit, with Gliclazide dosage stabilized at 60 mg per day). Although the patient had excellent glycaemic control already during insulin therapy, the therapeutic switch and especially the appropriate dosage of Gliclazide helped to further increase the Time In Range (TIR) and reduce the Time Above Range (TAR).

## 4 Discussion

In this case report, we describe a young patient initially diagnosed with non-ketoacidotic DM, later confirmed to have HNF4A-MODY. The diagnosis, which was suspected based on clinical features and confirmed by molecular testing along with functional analysis of a novel variant, had significant implications for the management of the patient.

Young patients presenting with a clinical onset of DM requiring insulin treatment are diagnosed as Type 1 Diabetes Mellitus (T1DM) while evidence of the presence of autoimmune antibodies is investigated. The absence of pancreatic autoantibodies, a low or no insulin requirements for longer than 5 years after the diagnosis and the absence of signs of Type 2 Diabetes Mellitus (T2DM) (marked obesity, acanthosis nigricans) prompts suspicion of monogenic diabetes, particularly in cases with a family history of DM in one parent or first-degree relative (15).

Maturity-Onset Diabetes of the Young 1 (MODY1) is a rare and hereditary monogenic form of diabetes due to heterozygous mutations in the *HNF4A* gene. It encodes a transcription factor crucial for the development and function of pancreatic β-cells, which are responsible for insulin secretion in response to glucose (16). The gene is involved in a well-coordinated network of transcriptional regulation, comprising several genes such Glucose Transporter 2 (GLUT2/SLC2A2), which facilitates the glucose uptake into β-cells; enzymes involved in the downstream glycolytic pathway (such as GCK, PKLR, GAPDH); genes encoding channels central to the ATP-dependent pathway of insulin secretion (such as KCNJ11) (16–18). Moreover, HNF4A regulates insulin expression both directly, through the binding of consensus sequences in the promoter of the *INS* gene and indirectly through HNF1A transcription factor (19). Indeed, HNF4A and HNF1A form a cross-regulatory network, where each regulates the other's expression, highlighting their interconnected roles in β-cell function (19). It's important to note that HNF4A has many target genes beyond those directly involved in insulin secretion, as it plays a broad role in liver, kidney, and intestinal function as well (16, 20). Literature on MODY1 is limited and primarily focused on mutations defining the condition (21) and some clinical manifestations characterizing its onset (macrosomia, neonatal hypoglycaemia, and gestational diabetes), which were absent in our patient (22).

Sulfonylureas are the first therapeutic choice for MODY1. Switching from insulin to sulfonylureas is considered successful, without deterioration in glycaemic control, in the patients previously treated with insulin (23). Sulfonylureas reduce blood glucose levels by stimulating insulin secretion from pancreatic beta cells. Among sulfonylureas, Gliclazide is usually chosen for its lower risk of hypoglycaemia and its favorable route of administration, since it can be taken orally, once or twice a day depending on the formulation (short or slow release). In adults, the initial dose is 30 mg per day and can then be increased up to 120 mg per day. The interval between each dose increase should be at least 1 month. In children, the initial dose should be lower to avoid hypoglycaemic events. The most common side effect is hypoglycaemic crises in case of irregularities in meals, and especially, in case of missed meals. Moreover, attention should be paid to patients with conditions of liver or kidney failure, malnutrition, imbalance between exercise and carbohydrate intake or endocrine dysfunctions (involving thyroid, pituitary and adrenal glands, among others). In absence of hypoglycaemia, patients maintain low-dose sulfonylureas (e.g., 20–40 mg gliclazide daily) for decades (22).

The benefits of this treatment extend beyond improved glycaemic control offering a better quality of life, particularly important for younger patients. This report underlines how integrating clinical and genetic diagnoses can contribute to precision medicine, enabling targeted treatment, personalized follow-up, and genetic counseling for both the patient and the family members.

## Data Availability

The data presented in the case report are deposited in publicly available IT systems of the hospital. Data shared are in accordance with the ethical consent provided by participants on the use of confidential/identifiable human data. The publication of such data does not compromise the anonymity of the participants or breach local data protection laws.
