# Supplementary material for: Case Report: Beyond type 1 diabetes: a case of delayed MODY1 diagnosis and successful transition to sulfonylurea therapy
Source: Front Med (Lausanne). 2025 May 23;12:1590935. doi: 10.3389/fmed.2025.1590935 (PMC12141343; doi:10.3389/fmed.2025.1590935)
Supplement: Supplementary file 1 [file Table_1.docx]

**Supplementary Table 1.**

List of the genes analysed for the molecular diagnosis.

| **N.** | **OMIM ID** | **Gene symbol** |
| --- | --- | --- |
| 1 | 600509 | *ABCC8* |
| 2 | 607358 | *AIRE* |
| 3 | 606844 | *ALMS1* |
| 4 | 604299 | *APPL1* |
| 5 | 107777 | *AQP2* |
| 6 | 300538 | *AVPR2* |
| 7 | 209901 | *BBS1* |
| 8 | 191305 | *BLK* |
| 9 | 611507 | *CISD2* |
| 10 | DIAPH1 | *DIAPH1* |
| 11 | 600576 | *GATA4* |
| 12 | 601656 | *GATA6* |
| 13 | 138079 | *GCK* |
| 14 | 121011 | *GJB2* |
| 15 | 610192 | *GLIS3* |
| 16 | 138130 | *GLUD1* |
| 17 | 601609 | *HADH* |
| 18 | 142410 | *HNF1A* |
| 19 | 189907 | *HNF1B* |
| 20 | 600281 | *HNF4A* |
| 21 | 147730 | *IL2RA* |
| 22 | 176730 | *INS* |
| 23 | 147670 | *INSR* |
| 24 | 600937 | *KCNJ11* |
| 25 | 603301 | *KLF11* |
| 26 | 606453 | *LRBA* |
| 27 | 605283 | *MAGEL2* |
| 28 | 601724 | *NEUROD1* |
| 29 | 605290 | *OPA1* |
| 30 | 606580 | *OPA3* |
| 31 | 167413 | *PAX4* |
| 32 | 607108 | *PAX6* |
| 33 | 600733 | *PDX1/IPF1* |
| 34 | 300039 | *POU3F4* |
| 35 | 612659 | *RFX6* |
| 36 | 602329 | *SEL1L* |
| 37 | 608937 | *SH2B1* |
| 38 | 182381 | *SLC5A2* |
| 39 | 608160 | *SOX9* |
| 40 | 610928 | *SOX17* |
| 41 | 600555 | *STAT1* |
| 42 | 102582 | *STAT3* |
| 43 | 604260 | *STAT5B* |
| 44 | 612988 | *TMEM126* |
| 45 | 606201 | *WFS1* |
